# The HLA Region and Autoimmune Disease: Associations and Mechanisms of Action

**DOI:** 10.2174/138920207783591690

**Published:** 2007-11

**Authors:** S.C.L Gough, M.J Simmonds

**Affiliations:** Division of Medical Sciences, University of Birmingham, Institute of Biomedical Research, Birmingham, B15 2TT, UK

**Keywords:** Genes, autoimmunity & HLA

## Abstract

The HLA region encodes several molecules that play key roles in the immune system. Strong association between the HLA region and autoimmune disease (AID) has been established for over fifty years. Association of components of the HLA class II encoded *HLA-DRB1-DQA1*-*DQB1* haplotype has been detected with several AIDs, including rheumatoid arthritis, type 1 diabetes and Graves’ disease. Molecules encoded by this region play a key role in exogenous antigen presentation to CD4+ Th cells, indicating the importance of this pathway in AID initiation and progression. Although other components of the HLA class I and III regions have also been investigated for association with AID, apart from the association of HLA-B*27 with ankylosing spondylitis, it has been difficult to determine additional susceptibility loci independent of the strong linkage disequilibrium (LD) with the HLA class II genes. Recent advances in the statistical analysis of LD and the recruitment of large AID datasets have allowed investigation of the HLA class I and III regions to be re-visited. Association of the HLA class I region, independent of known HLA class II effects, has now been detected for several AIDs, including strong association of *HLA-B* with type 1 diabetes and *HLA-C* with multiple sclerosis and Graves’ disease. These results provide further evidence of a possible role for bacterial or viral infection and CD8+ T cells in AID onset. The advances being made in determining the primary associations within the HLA region and AIDs will not only increase our understanding of the mechanisms behind disease pathogenesis but may also aid in the development of novel therapeutic targets in the future.

## INTRODUCTION

Autoimmune diseases (AIDs) including type 1 diabetes (T1D), multiple sclerosis (MS), rheumatoid arthritis (RA), Graves’ disease (GD), ankylosing spondylitis (AS) and systemic lupus erythematosus (SLE) affect over 4% of the western population and involve the generation of autoantibodies against self antigen/s. In many cases lymphocytic infiltration of a target organ/s and autoimmune destruction is also observed. These diseases are often chronic and debilitating and, although treatments are available, many are inadequate [1] providing the impetus for a greater understanding of the disease mechanisms and the development of novel therapeutic agents. Antigen presentation and T cell activation appear to be key to triggering an autoimmune response [2] prompting investigation of many genes within this pathway for association with AID including several within the major histocompatibility complex (MHC). 

**Fig. (1):**
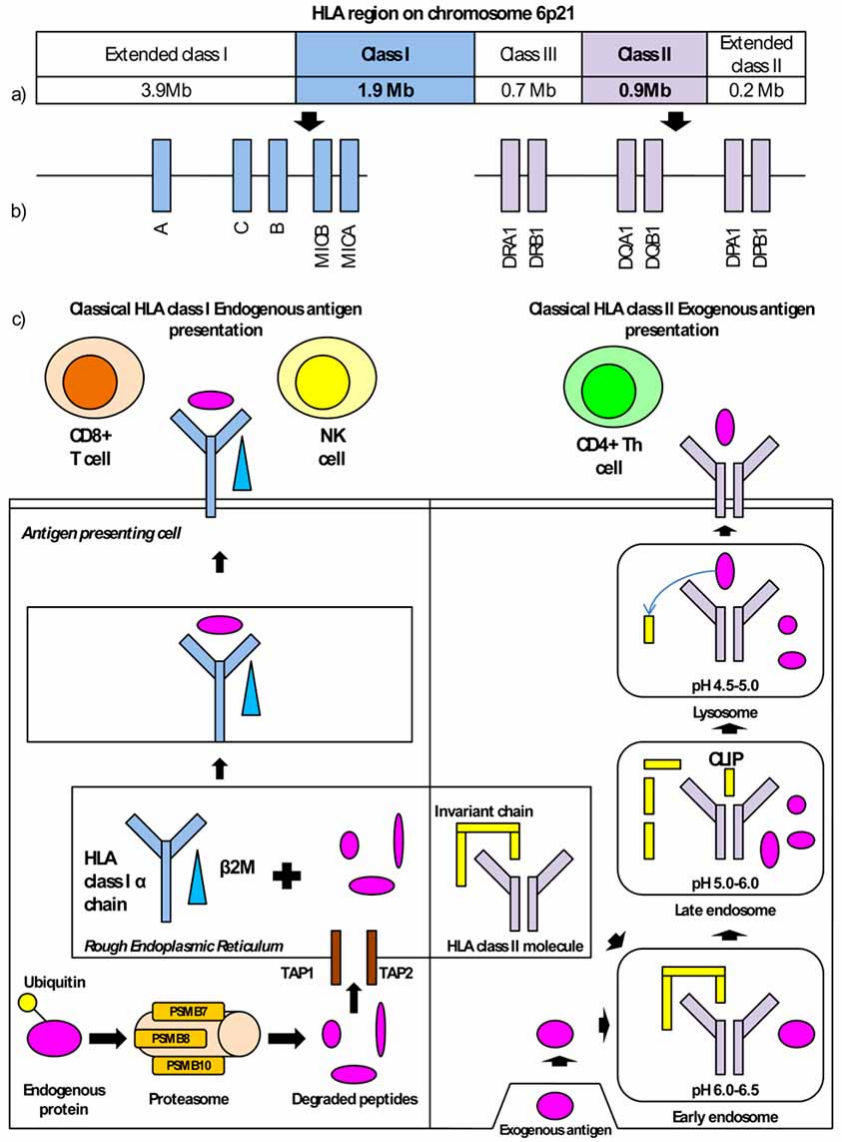
**a)** Diagrammatic representation of the HLA region on chromosome 6p21, **b**) More detailed diagrams of common AID genes within the HLA class I and class II region **c**) Representation of endogenous antigen presentation by HLA class I classical molecules (including HLA-A, -B and –C). Endogenous antigen generated in the cytosol is degraded within the proteasomes and then transported into the rough endoplasmic reticulum (RER) through the TAP1/TAP2 complex. Antigen is then bound by HLA class I molecules in connection with β2M and then once bound the molecule is exported to the cell surface for recognition by CD8+ T cells and natural killer (NK) cells. Exogenous antigen presentation by HLA class II classical molecules (including HLA-DR and –DQ) is also represented. Exogenous antigen is imported into the cell and then enters the endosytic pathway (encompassing the early endosome, late endosome and lysosome) where the antigen is degraded. At the same time HLA class II molecules complexed with the invariant chain move from the RER where they are synthesised to the endocytic pathway. As the HLA class II/Invariant chain complex moves into the increasing more acidic compartment of the endocytic pathway the in-variant chain is digested, leaving only CLIP bound. The CLIP is then replaced with degraded antigen and then the HLA class II mole-cule/antigen complex is exported to the surface of the cell for presentation to CD4+ T helper (Th) cells. PSMB = Proteasome subunit B –type, TAP = Transporters associated with antigen presentation, β2M = β2 Microglobulin, CLIP = class II associated invariant chain.

The MHC, also known in humans as the human leukocyte antigen (HLA) region, encompasses 7.6Mb on chromosome 6p21 and is the most gene dense region within the human genome encoding 252 expressed loci [3] including several key immune response genes. The region can be subdivided into the extended class I, classical class I, classical class III, classical class II and extended class II regions (Fig. **1**) and contains the largest degree of polymorphism within the genome [4]. It also exhibits the most dense linkage disequilibrium (LD) [5], extending up to 540kb, which compares with distances of between 1-173kb seen in the rest of the genome [6, 7]. The extent of LD within the region has proved challenging when trying to tease out the exact location of etiological variants. Most studies have focussed on the role of HLA class II encoded HLA-DR and –DQ molecules, which present exogenous antigens for recognition by CD4+ T helper (Th) cells. Strong associations with almost all AIDs have been detected (see Table **1**).

## ASSOCIATION OF THE HLA CLASS II REGION WITH AID

Early experiments investigating association of HLA-*DR* and -*DQ* with AID in T1D indicated a strong association of the DRB1*03 and DRB1*04 alleles [8-10]. A stronger association with the *DQB1* locus alone was reported when an allele encoding aspartic acid (Asp) at position β57 of DQB1 was found to be associated with resistance to T1D, while an allele encoding a neutral residue, such as alanine (Ala) or serine (Ser) at position β57 conferred susceptibility [11-13]. This molecule forms a critical residue in peptide binding pocket nine (P9) of the DQB1 binding pocket involved in antigen presentation and T cell receptor (TCR) interaction. It’s carboxylate group forms a salt bridge with arginine (Arg) at position α57 of the DQA1 chain that stabilizes the heterodimer between the DQA1 and DQB1 chains [13]. The presence of Asp at this position could alter the stability of the molecule and/or antigen presenting repertoire, thus making the molecule more prone to binding autoreactive antigens. Although this may provide an interesting mechanism for association of the HLA class II region with T1D, further studies have demonstrated a potential role for other residues in DQB1 binding pocket P9 and DRB1 binding pockets P1 and P4 in disease susceptibility [13]. 

Association with RA is only seen at *DRB1, *with other associations with *DQB1* [14] almost certainly the result of LD with *DRB1* [15, 16]. Originally association was attributed to DRB1*04 [17] but other associations have subsequently been reported. Work by Gregerson, *et al*, showed that association of *DRB1* alleles (DRB1*0101, DRB1*0102, DRB1*0401, DRB1*0404, DRB1*0405, DRB1*0408, DRB1*1001 and DRB1*1402) [18, 19] was due to similarities within the DRB1 peptide domain at positions β70 through to β74, with these alleles containing similar sequences (QKRAA/QRRAA/RRRAA) termed the ‘shared epitope’ [18]. Amino acid changes within the shared epitope have been postulated to differentiate the predisposing alleles from the protective DRB1*0103, DRB1*07, DRB1*1201, DRB1*1301 and DRB1*1501 alleles [2, 15, 20]. Recent work on the shared epitope has suggested a new model, whereby the shared epitope only consists of positions β72-β74, and rather than being essential for disease susceptibility, positions β70 and β71 have been proposed to modulate association [21, 22]. Further verification of this hypothesis, re-defining the shared epitope, is required. 

Association of DRB1 position β74 of the share epitope is not unique to RA. In GD, comparison of the predisposing DRB1*03 and protective DRB1*07 molecules showed DRB1*03 containing a positively charged Arg at β74 and DRB1*07 containing a non-charged polar glutamine (Gln) at this position. Position β74 has also been shown to differentiate between the lower risk DRB1*0403 and *0406 T1D alleles, which contain a negatively charged glutamic acid (Glu), compared to the high risk DRB1*0401 allele which contain a non-charged polar Ala [23-26]. Interestingly the DRB1*07 haplotype is also protective against T1D and RA [27-29]. Amino acid (74 spans several binding environments involved in autoantigen binding/presentation and T cell antigen receptor docking and interaction [30], suggesting that changes within peptide and TCR binding could determine which amino acids are presented to the immune system. 

Further work on *DRB1* and *DQB1* showed that there was LD between these molecules and, along with *DQA1*, they were found to form part of extended haplotypes DRB1*03-DQB1*02-DQA1*0501 (DR3) and DRB1*04-DQB1*0302-DQA1*0301 (DR4) associated with T1D [2, 31]. Overlap between HLA class II associations has been seen between AIDs, with DR3 also being associated with GD, SLE and HT, therefore, being termed the ‘autoimmunity haplotype’ (See Table **1** for list of common AID HLA class II associations). DR4 is also associated with other AIDs, including HT. Statistical modelling techniques, including logistic regression analysis, have been employed to determine which components of the DRB1-DQB1-DQA1 haplotype contain the etiological variants associated with AID. In GD this approach was successful in determining that *DQB1* was unable to explain association with disease, narrowing down the location of the etiological variant within the class II region to either *DRB1* or *DQA1 *[32]. 

Sharing of susceptibility loci between AIDs is not unique to the HLA region with variants within *CTLA-4, PTPN22* and* CD25* also being shared [2, 33-35]. There are however distinct differences between the AIDs, with the DR15 (DRB1*15- DQB1*0602-DQA1*0102) haplotype strongly predisposing for MS [12, 36], whilst protecting against T1D. The observation, therefore, of a number of different *DR* and/or *DQ* associations, with AID suggests that there are common and unique disease pathways for each AID which may, in part, be explained by the presentation of different disease-specific autoantigens [1]. 

## HLA CLASS II DISEASE MECHANISMS

The role played by the HLA-DR/-DQ molecules in exogenous antigen presentation to CD4+ Th cells helps to explain their association with several AIDs (see Fig. **1**). Exogenous peripheral antigens are internalized *via *antigen presenting cells (APC) and are degraded into 13-18 amino acid residue peptides, preferentially bound by HLA class II molecules, in the increasingly acidic compartments of the endocytic pathway. HLA class II molecules are synthesized in the rough endoplasmic reticulum (RER) where they associate with the invariant chain (Ii) to prevent endogenous peptide binding. The HLA class II molecule is then routed to the endocytic pathway, where Ii is degraded, leaving a short fragment of the Ii class II-associated invariant chain peptide (CLIP) bound, which is then exchanged for peptide [37]. The HLA class II peptide complex is then transported to the cell surface for recognition by CD4+ Th cells, which determine whether an immune response is mounted. If an immune response is mounted CD4+ Th cells activate naive B cells to produce antibodies, or in the case of self-antigens autoantibodies, and aid in macrophage recruitment. Activated autoreactive CD4+ Th cells against a variety of exogenous autoantigens, including pancreatic beta cells, thyroid components and myelin, have been detected in TID, GD and MS respectively [38], suggesting a role in disease susceptibility.

Several non-mutually exclusive mechanisms have been proposed to explain association of DR/DQ with AIDs:

- Variation in binding groves of associated DR/DQ molecules could lead to preferential presentation of only a specific limited set of self peptides or low affinity self peptides may allow autoreactive T cells to escape tolerance and enter the periphery. This could affect thymic T cell education, causing incomplete thymic tolerance and a Th and T regulatory (Treg) cell population that does not recognize all self molecules.
- Polymorphic residues of the TCR-exposed surfaces of DR/DQ could select autoreactive T cells or fail to select a good Treg population. It has been proposed that the protective effects seen with specific HLA molecules could be due to the generation of Treg cells that are able to keep autoreactive T cells in check and prevent autoimmunity [39]. Studies in RA have shown Treg populations to be compromised [40], suggesting that RA could be associated with a failure to protect rather than a predisposition to disease.
- Promiscuous restriction where some TCRs will use a series of different restriction elements to bind to and, therefore, interact with a wider variety of different peptides.
- Preferential binding in DR/DQ heterozygous subjects could be occurring through epitope stealing by one HLA molecule over another which, depending on TCR restriction of CD4+ Th cells, could affect whether an immune response is mounted [41].
- Presentation of endogenous antigens by HLA class II. Although class II molecules traditionally present exogenous antigens and class I present endogenous antigens (see Fig. **1**), this system is not absolute and presentation of endogenous antigens by class II and exogenous antigens by class I can be seen [42, 43]. This could alter how these antigens are presented to the immune system and the response triggered.

These hypotheses suggest a series of potential mechanistic pathways by which the HLA class II molecules could be involved in disease onset by altering the Th and Treg cell repertoire or through changes in how the antigen is recognized in the periphery.

## EVIDENCE FOR ROLE OF HLA CLASS I IN DISEASE

Association of HLA class I molecules, in particular HLA-A, -B and –C, has been detected for several AIDs. HLA-B*27 has been known for many years to predispose to AS, and is present in over 90% of European AS patients [44]. Of the 31 HLA-B*27 suballeles detected to date [45] the B*2701, B*2704 and B*2705 alleles are strongly predisposing for AS [45, 46], whereas the B*2706 and B*2709 alleles are protective [46, 47]. Interestingly, the B*2705 only differs from the B*2709 at amino acid residue 116, *via *an Asp or histidine (His), respectively, which is known to alter peptide specificity and T cell recognition [45, 48]. Although *HLA-A, -B* and *–C* are located in the HLA class I region, they exhibit LD with the HLA class II encoded DR/DQ haplotypes. The presence of this strong LD with the HLA class II region has made it difficult to identify independent effects for the class I region with any AIDs except AS which exhibits no strong class II associations, although further confirmation of potential HLA class II effects in AS is needed [49]. The utilization of larger datasets and the development of more advanced statistical analysis, has enabled re-investigation of the HLA class I region as an independent general AID locus.

Work in T1D identified a microsatellite D6S2223 4.9kb telomeric of DQ in the extended class I region, as being associated with a reduction in the risk conferred by the DR3 extended haplotype on disease [50], with further work narrowing the association to a region encompassing the class III and *HLA-B/-C* gene regions [51]. Work in the NOD mouse also suggested the presence of independent risk factors within the MHC, independent of the MHC class II [52], with further genetic studies in humans confirming the presence of HLA class II independent T1D susceptibility loci within the class I region [53, 54]. More recently, screening of a total of 1729 polymorphisms across the whole HLA region in several independent Caucasian T1D datasets, revealed evidence of a secondary peak of association for T1D independent of known *DQB1 *and *DRB1 *effects, due to the HLA-B locus (P_combined_ = 2.01 x 10^-19^) with the B*39 allele consistently associated with T1D and some evidence for an effect of B*18 [55]. Association of *HLA-A *was also detected (P_combined_ = 2.35 x 10^-13^) [55], with predisposing effects for A*24 and protective effects for A*01, A*11 and A*31 identified. 

The presence of additional susceptibility loci within the HLA class I gene region has also been detected for MS. HLA class II independent association of two microsatellite markers (D6S1683 and D6S265) in close proximity to the HLA class I region was reported [56]. Association studies within a small Swedish dataset demonstrated a predisposing effect for the A*0301 allele and a protective effect for the A*0201 allele in patients with MS [57], independent of the known DR15 association, with further studies confirming the protective effect of A*0201 [58]. A recent large MS case control study further confirmed an independent protective effect of A*0201 (P = 7 x 10^-12^), although failed to detect an independent association for A*0301 [59]. Using a combination of microsatellite markers, tagging single nucleotide polymorphisms (SNPs) and classical HLA typing, the International MS genetics consortium detected an independent protective effect for C*05 (P = 3.3 x 10^-5^), in both a family and case control dataset [60]. Although this study did not find any support for independent *HLA-A* effects, they could not rule out an effect at this locus [60]. Interestingly when *HLA-B* and *–C *were screened for association with GD and logistic regression analysis applied to see if these effects were independent of known HLA-DR/-DQ affects, *HLA-C*, and to a lesser extent *HLA-B*, produced stronger association (P = 1.20 x 10^-20^ and P = 1.54 x 10^-6^, respectively) with disease than the previously established HLA class II effect, suggesting a key role in disease susceptibility [32]. 

## HLA CLASS I DISEASE MECHANISMS

HLA class I molecules present endogenous antigens, such as those derived from viruses and intracellular bacteria, for recognition by the immune system (see Fig. **1**). This process involves ubiquitination of endogenous cytosolic proteins and then degradation into short 8-16 amino acid peptides, optimal for HLA class I binding. These are subsequently transported into the RER where they bind HLA class I molecules combined with β2 microglobulin (β2M), before exiting the RER and being transported to the cell surface [37]. HLA class I presented antigen is then recognized by CD8+ T cells and natural killer (NK) cells. Once CD8+ T cells become activated, functional effector T lymphocytes (CTLs) are produced which possess lytic capabilities and also play a role in generating CD8+ T memory cells, acting as part of both the innate and adaptive immune responses. Activated NKs act before clonal expansion and differentiation of CD8+ T cells and compliment the CTL response. They act as one of the first lines of innate immune defence by producing lymphokines, including interferons, which aid in the recruitment of additional cells to the site of inflammation [61] and also produce cytokines and chemokines that have a cytolytic activity aiding cell destruction. 

There is increasing evidence for a role of CD8+ T cells in AID. Knocking out MHC class I expression in the SLE prone mouse suggested an important role for CD8+ T cells in autoimmunity as it failed to develop SLE [62]. Further models suggested a key role for CD8+ T cells in AID progression, rather than in initiation [63, 64]. Recently CD8+ T cells have been detected in the peripheral blood, cerebrospinal fluid and lesions of MS sufferers and were shown to destroy multiple central nervous system cell substrates, including myelin peptides [65, 66]. Suppressor CD8+ T cells have also been found to be decreased in relapsing MS subjects [66]. Work in T1D has demonstrated CD8+ specific T cell responses and a potential role for defective CD8+ T suppressor cells [67-69] with CD8+ T cells responses also shown to be important in SLE and GD [70].

Several hypotheses have been suggested to explain how variation in HLA class I genes could trigger autoimmunity. HLA class I molecules play a role in presenting endogenous antigens, including those derived from viruses and/or bacteria, which have been proposed to be key environmental triggers for AID (see Table **2** for a list of proposed viral triggers of AID). Viral/bacterial antigens may trigger AID through molecular mimicry and *via *acting as superantigens. Molecular mimicry occurs when microbial antigens that are sufficiently similar to self-antigens activate autoreactive T-cells that can cross react with self antigen triggering autoimmunity. Viral/bacterial antigens could also act as superantigens, producing a strong non-specific immune response that then cross reacts attacking other tissues in the body [71, 72]. Viruses can also alter HLA class I and II expression, potentially leading to greater antigen presentation to CD8+ T cells, with certain alleles more prone to viral/bacteria manipulation [73]. During viral infection soluble HLA levels, involved in regulating the immune response, have also been shown to be increased in RA patients, the level of which is dependent on HLA allele present [74, 75]. If confirmed these mechanisms could also help validate the role of viral/bacterial infection in AID onset. 

HLA class I molecules could also be associated with AID due to their role in inhibiting NK cell activity. NK cell cytotoxic activity is controlled by a balance between activating and inhibitory receptors on their surface [76]. Activation signals are blocked by inhibitory signals produced through interaction of killer immunoglobulin-like receptors (KIR) with a variety of HLA class I molecules loaded with peptide [76, 77]. If HLA class I expression is normal then NK cell mediated destruction is inhibited. For example, there are two sets of KIRs, 2DL1 and 2DL2/2DL3 which recognize specific sets of HLA-C molecules, group C1 molecules (containing C*02, *04, *05 and *06) and group C2 molecules (containing C*01, *03, *07 and *08), respectively [78]. Groups C1 and C2 are distinguished by the presence of Ser at position 77 and asparagine (Asn) at position 80 of the (1 helix or Asp at position 77 and a lysine (Lys) at position 80, respectively. KIR/HLA-C interactions can be altered by peptide loading and presentation by HLA-C [79, 80], which could suggest that interaction seen in GD and MS of the associated HLA-C molecules with a given autoantigen/s could be affecting KIR binding and that this interaction between KIRs and HLA-C could play a role in autoimmune onset [81, 82]. Variation in the interaction of other molecules that bind to HLA class I and monitor HLA class I expression, including leukocyte Ig-like receptors (LIRs), members of the Ly49 family (Ly49) and the CD94/NKG2 family of receptors, could also provide another mechanism by which HLA class I could lead to AID [76, 77, 83]. 

Non-viral mechanisms have also been proposed including:

- protein misfolding causing specific molecules to accumulate in the RER, where they are degraded, potentially causing a pro-inflammatory unfolded protein stress response or misfolded proteins themselves to become autoantigenic [48].
- Conversion of HLA class I molecules into peptides which could then be presented by HLA class II molecules to the immune system and an immune response mounted as has been proposed in AS with B*27 [43].
- Peptide binding and presentation by specific HLA molecules as suggested earlier for HLA class II molecules with mechanisms including selection of antigen specific CD8+ T cells during thymic education, and positive or negative selection of a strong CD8+ T suppressor population [84].

## ARE THERE ANY OTHER HLA ASSOCIATIONS?

Once the classical HLA class II and/or class I effects have been taken into account it becomes increasingly difficult to detect further associations, suggesting that even larger datasets and new techniques to cover more variation within this region are required [55]. It should be noted that although HLA class II and class I appear to play a role in almost all AIDs, in some, the associations may lie outside of the classical loci. Strong association of HLA-C with psoriasis was detected and presumed to be the etiological variant, until further studies suggested that the true etiological variant actually lies 60kb outside of the HLA-C molecule [85, 86]. Although this review has focused on the classical HLA class I and II associations which have shown strong and consistent association with AID, several other genes within the HLA region may be acting as independent risk factors for AID (Table **3**). Further work is required to determine if these genes are true AID etiological variants [87, 88], using techniques, for example, which utilise the development of tag SNPs to screen the HLA region [89].

## CONCLUSIONS

With our increased understanding of the complexity within the HLA region and the development of new statistical methodologies to help decipher the unique LD structure therein, progress has been made in starting to disentangle the associations present [89, 90], providing valuable insights into AID mechanisms. Novel therapeutic treatments based on these discoveries have already been proposed. The development of protease inhibitors designed to alter the antigen presenting properties of HLA molecules, by blocking the presentation of potentially auto-antigenic peptides, could direct the immune system away from a particular antigenic motif and in turn the autoimmune disease process [37]. Although clinical use may be some way off, continued research on the whole HLA region is vital to increase our understanding of the key mechanisms behind AID and ultimately the provision of new therapeutic targets.

## Figures and Tables

**Table 1:** Summary of the Major Associations Within the HLA Class II and Class I Region with Common Autoimmune Diseases

HLA Class II Effects		AUTOIMMUNE DISEASE	HLA Class I Effects	
Predisposing	Protective		Predisposing	Protective
◊	◊	**Ankylosing spondylitis**	B*2701 B*2704 B*2705	B*2706 B*2709
DR3 DRB1*08	DR7	**Graves’ disease**	C*07 B*08	C*16 C*03 B*44
DR4 DR3	DR7	**Hashimoto’s thyroiditis**	◊	◊
DR3	◊	**Myasthenia Gravis**	◊	◊
DR3	◊	**Addison’s disease**	◊	◊
Shared epitope = DRB1*0101 DRB1*0102 DRB1*0401 DRB1*0404 DRB1*0405 DRB1*0408 DRB1*1001 DRB1*1402	DRB1*0103 DRB1*07 DRB1*1201 DRB1*1301 DRB1*1501	**Rheumatoid arthritis**	◊	◊
DQ2 DQ8	◊	**Celiac disease**	◊	◊
DR15	DR14	**Multiple sclerosis**	C*05 C*15	C*01
DR3 DR4 DQB1 position β57	DR15 DR14	**Type 1 diabetes**	B*39 B*18 A*24	A*01 A*11 A*31
DR3 DR8 DR15	◊	**Systemic lupus erythematosus**	◊	◊

Both allelic and haplotype associations have been shown.

◊ = Further studies needed to determine association.DR3 = DRB1

* 03-DQB1*02-DQA1*0501, DR4 = DRB1*04-DQB1*0302-DQA1*0301, DR7 = DRB1*07-DQB1*02-DQA1*02, DR14 = DRB1*14-DQB1*06-DQA1*0102, DR15 = DRB1*15-DQB1*06-DQA1*01, DQ8 = DQB1*0302 and DQ2 = DQB1*0201.

**Table 2:** List of Proposed Viral Triggers for Autoimmune Disease

Virus	Virus Family	Symptoms Caused by Virus	Autoimmune Disease/s
**Adenovirus **	*Adenoviridae *	Upper respiratory infections	GD [91]
**Coxsachie B virus **	*Picornaviridae *	Gastrointestinal infections and in more extreme cases myocarditis (inflammation of the heart) and pericarditis (inflammation of the sack surrounding the heart)	T1D [92-95]
**Hepatitis B **	*Hepadnaviridae*	Liver inflammation	SLE [96, 97]
**Hepatitis C **	*Hepadnaviridae*	Liver inflammation	MG [98, 99], GD [100, 101], T1D [102, 103], SLE [104]
**Herpes simplex virus 1 & 2 **	*Herpesviridae *	Blisters in the skin/lips/mouth/genitals and then infection become latent for future re-activation	MS [105, 106]
**Herpes simplex virus 3 (Varicella Zoster Virus)**	*Herpesviridae*	Causes chicken pox during initial infection and upon reactivation can cause shingles	MS [105, 107]
**Herpes simplex virus4 (Epstein Barr Virus)**	*Herpesviridae*	Often asymptomatic but can cause infectious mononucleosis result-ing in fever, sore throat, muscle soreness and fatigue	RA [108, 109], SLE [108], MS [108, 110, 111]
**Herpes simplex virus 5 (Cytomegalovirus) **	*Herpesviridae *	Latent infection that in healthy individuals causes limited symptoms but can be more detrimental in immuno-compromised individuals	SLE [112, 113], MS [114]
**Herpes simplex virus 6**	*Herpesviridae*	Infects almost all children causing a rash before becoming latent	MS [105, 115, 116]
**Human Coronavirus (HCoV) **	*Coronaviridae*	Upper respiratory and gastrointestinal tract infection	MS [117]
**Human Foamy Virus **	*Retroviridae *	Asymptomatic	GD [118, 119], RA [120], SLE [120], MG [121]
**Human immunodeficiency virus (HIV) **	*Retroviridae *	Leads to low levels of CD4+ T cells, resulting in a compromised immune system, making the person susceptible to opportunistic infection	SLE [122, 123], MG [123]
**Human T cell leukaemia virus (HTLV) **	*Retroviridae *	T cell leukaemia and T cell lymphoma	GD [124, 125], SLE [126]
**John Cunningham (JC) virus **	*Polymaviridae *	Asymptomatic unless in immuno-compromised patients where is can cause progressive multifocal leukoencephalopathy and other diseases	MS [127-129]
**Parvovirus B19 **	*Paroviridae *	Causes childhood exanthema (a widespread rash)	HT [130], RA [131, 132], T1D [132, 133], GD [132], MS [134], MG [135]
**Rotavirus **	*Reoviridae *	Infection of the gastrointestinal tract	T1D [136]
**Transfusion transmitted (TT) virus **	*Circoviridae *	Asymptomatic	RA [137]

GD = Graves’ disease, T1D = type 1 diabetes, RA = rheumatoid arthritis, SLE = systemic lupus erythematosus, MS = multiple sclerosis, MG = Myesthenia Gravis, HT = Hashimoto’s thyroiditis.

**Table 3:** Genes within the HLA Region which have been Proposed to Contribute to Autoimmune Disease (AID) Susceptibility Independently of Known HLA Class I and Class II Association

Gene	Region	Function	AIDs Proposed to be Linked to these Genes
*BAT family members*	Class III	Believed to play a role in negatively regulating inflammation.	RA [138-140]
*BTNL2 *	Class II	BTNL2 has structure features including an IgC domain which it shares with CD80/CD86, which act as co-stimulatory receptors for T cell activation, including interacting with CTLA-4, and other molecules involved in T cell inhibition including B7-RP.	GD [141]
*Complement component C4 *	Class III	Plays a role in both the classical and lectin pathways of complement activation which leads to several outcomes including activating inflammation, secretion of immunoregulatory molecules that fine tune the immune response, clearance of immune complexes, opsoniza-tion (aiding antibodies to enhance the ability of phagocytic cells to attack bacteria) and lysis of bacteria, viruses and cells.	SLE [142-149]
*MICA/MICB *	Class I	Stress induced molecule found on the surface of epithelial cell lines which is similar to the classical class I molecules but does not associate with β2M or bind antigen.	T1D [150-154], MS [155], RA [156, 157]
*TNFα *	Class III	A multifunctional cytokine secreted by macrophages and T lymphocytes with wide-ranging biological effects including protection from infection, surveillance against tumors and stimulation of inflammatory responses.	RA [158], GD [159], SLE [160]

RA = rheumatoid arthritis, GD = Graves’ disease, SLE = systemic lupus erythematosus, T1D = type 1 diabetes, MS = multiple sclerosis.
